# Habitat-based MRI radiomics for enhanced parkinson’s diagnosis

**DOI:** 10.1038/s41598-026-37923-y

**Published:** 2026-02-03

**Authors:** Yuan-Zhe Li, Yi Wang, Chi Cai, Si-Qing Cai, Yi-Fan Guo, Shu-Fen Liu, Chun-Nuan Chen, Tie-Qiang Li

**Affiliations:** 1 School of Medical Imaging, Fujian Medical University, Fuzhou, China; 2 Center of Radiology, The 2nd Affiliated Hospital of Fujian Medical University, Quanzhou, China; 3 Department of Radiology, The 1st Affiliated Hospital of Zhejiang Chinese Medical University, Hangzhou, China; 4 Department of Neurology, The 2nd Affiliated Hospital of Fujian Medical University, Quanzhou, China; 5 Department of Medical Radiation and Nuclear Medicine, Karolinska University Hospital, Karolinska Institutet, Stockholm, 14186 Sweden

**Keywords:** Parkinson’s disease (PD), MRI, Habitat-based radiomics, Diagnosis, Machine learning, Diseases of the nervous system, Diagnostic markers, Neurology

## Abstract

Early and accurate diagnosis of Parkinson’s disease (PD) remains a clinical challenge. This study explores the potential of habitat-based radiomics as a novel approach to improve PD detection using routine clinical MRI scans. We analyzed MRI data from 308 participants (173 PD patients and 135 healthy controls) to extract detailed features from segmented habitats in the caudate nucleus and putamen. Machine learning models, trained on habitat-based radiomic features, achieved a diagnostic accuracy exceeding 94%. This superior performance, compared to traditional radiomics, highlights the ability of habitat-based radiomics to capture subtle tissue heterogeneity associated with PD. Our findings suggest that habitat-based radiomics could be a valuable tool for early and accurate PD diagnosis, enabling timely intervention and improved patient outcomes.

## Introduction

Parkinson’s disease (PD) is a progressive neurodegenerative disorder marked by the loss of dopamine-producing neurons in the substantia nigra pars compacta, leading to characteristic motor symptoms such as tremor, rigidity, bradykinesia, and postural instability^1–4^, as well as non-motor symptoms including cognitive impairment, sleep disturbances, and depression. Early and accurate diagnosis of PD is essential to enable timely intervention and optimize disease management^5–7^.

Current diagnostic approaches primarily involve clinical assessments and imaging techniques like DaTscan^8,9^, which visualizes dopamine transporter levels in specific brain regions. While DaTscan and similar neuroimaging tools can support diagnosis by indicating dopamine system deficits, they often lack sufficient sensitivity and specificity, particularly in the early stages of PD^8–13^. This diagnostic gap has spurred efforts to identify more precise imaging biomarkers, with structural MRI^14–21^, functional MRI^22,23^, and diffusion tensor imaging^10–12^ being extensively explored.

Radiomics^4,21^, a quantitative approach to image analysis, has shown promise for extracting numerous imaging features related to texture, shape, and intensity from medical scans^17,24,25^. Combined with machine learning, radiomics offers a potential pathway to enhance the accuracy of PD diagnosis and prognosis. However, traditional radiomics typically analyzes predefined regions of interest (ROIs) as homogeneous entities^26–28^,^,^ extracting features from the entire ROI without accounting for spatial variations within the tissue. This approach may overlook critical subregional heterogeneity, such as localized neuronal loss or microstructural alterations, which are particularly relevant in neurodegenerative diseases like PD. For example, traditional radiomics might average out textural differences across the caudate nucleus or putamen, potentially missing focal changes that are indicative of early PD pathology.

Habitat-based radiomics addresses these limitations by segmenting ROIs into distinct sub-regions, or “habitats,” based on their unique imaging characteristics, such as intensity and texture variations. This method allows for a more granular analysis of tissue heterogeneity, capturing subtle differences within a region that may reflect disease-specific changes. For instance, in oncology, habitat-based radiomics has been successfully applied to characterize intratumoral heterogeneity in cancers such as glioblastoma and esophageal^29–34^. In these cases, segmenting tumors into habitats based on perfusion or metabolic activity has revealed distinct sub-regions associated with aggressive tumor behavior or treatment resistance, improving prognostic accuracy. Similarly, in PD, segmenting brain regions like the caudate nucleus and putamen into habitats could uncover localized patterns of neurodegeneration, such as variations in tissue density or microstructural integrity, that are not detectable by traditional radiomics. By analyzing these habitats separately, habitat-based radiomics provides a more nuanced understanding of disease-related changes, potentially leading to earlier and more accurate diagnosis^33–35^. While promising in oncology, its application in PD remains underexplored. This approach may offer valuable insights into the neurodegenerative processes in PD, potentially enabling earlier and more accurate diagnosis.

The advantages of habitat-based radiomics over traditional methods lie in its ability to model spatial complexity and provide interpretable insights into disease processes. For example, in breast cancer imaging, habitat-based radiomics has identified sub-regions with distinct vascular properties, enabling better prediction of treatment response compared to whole-tumor analysis^30^. In the context of PD, this approach could reveal specific sub-regions within the basal ganglia that exhibit early pathological changes, such as altered texture or intensity due to neuronal loss, which may be masked in traditional radiomic analyses. Furthermore, by integrating habitat-based features with machine learning, this approach can enhance diagnostic models by focusing on the most disease-relevant sub-regions, improving both sensitivity and specificity.

This study investigates the use of habitat-based radiomics to enhance PD diagnostic accuracy using routine clinical MRI scans. By segmenting the caudate nucleus and putamen into distinct imaging habitats and extracting a comprehensive suite of radiomic features from these habitats, we aim to develop a robust machine learning model capable of differentiating PD patients from healthy controls. Our hypothesis is that habitat-based radiomics will outperform traditional radiomics by capturing the spatial heterogeneity of neurodegenerative changes, thereby providing a more reliable and clinically actionable tool for early PD diagnosis.

## Results

### Demographics of the study groups

A total of 308 participants were enrolled from two medical centers affiliated with Fujian Medical University (173 PD patients and 135 HC subjects). The train cohort, recruited from the Li-Cheng center, included 133 PD patients and 102 HC participants. The validation cohort from the Dong-Hai center comprised 40 PD patients and 33 HC subjects. Demographic analysis revealed no significant differences in age or sex distribution between PD and HC groups within or between the cohorts, ensuring a balanced and comparable dataset. T1-weighted (T1W) and T2-weighted (T2W) MRI data were acquired using different scanners and settings across the centers. This contributes to a generalizable dataset, reflecting real-world clinical applications where equipment variations are common. Volumes of interest (VOIs) within the brain for the caudate nucleus and putamen were meticulously delineated using the ITK-SNAP software.

### Habitat segmentation

**Fig. 1:**
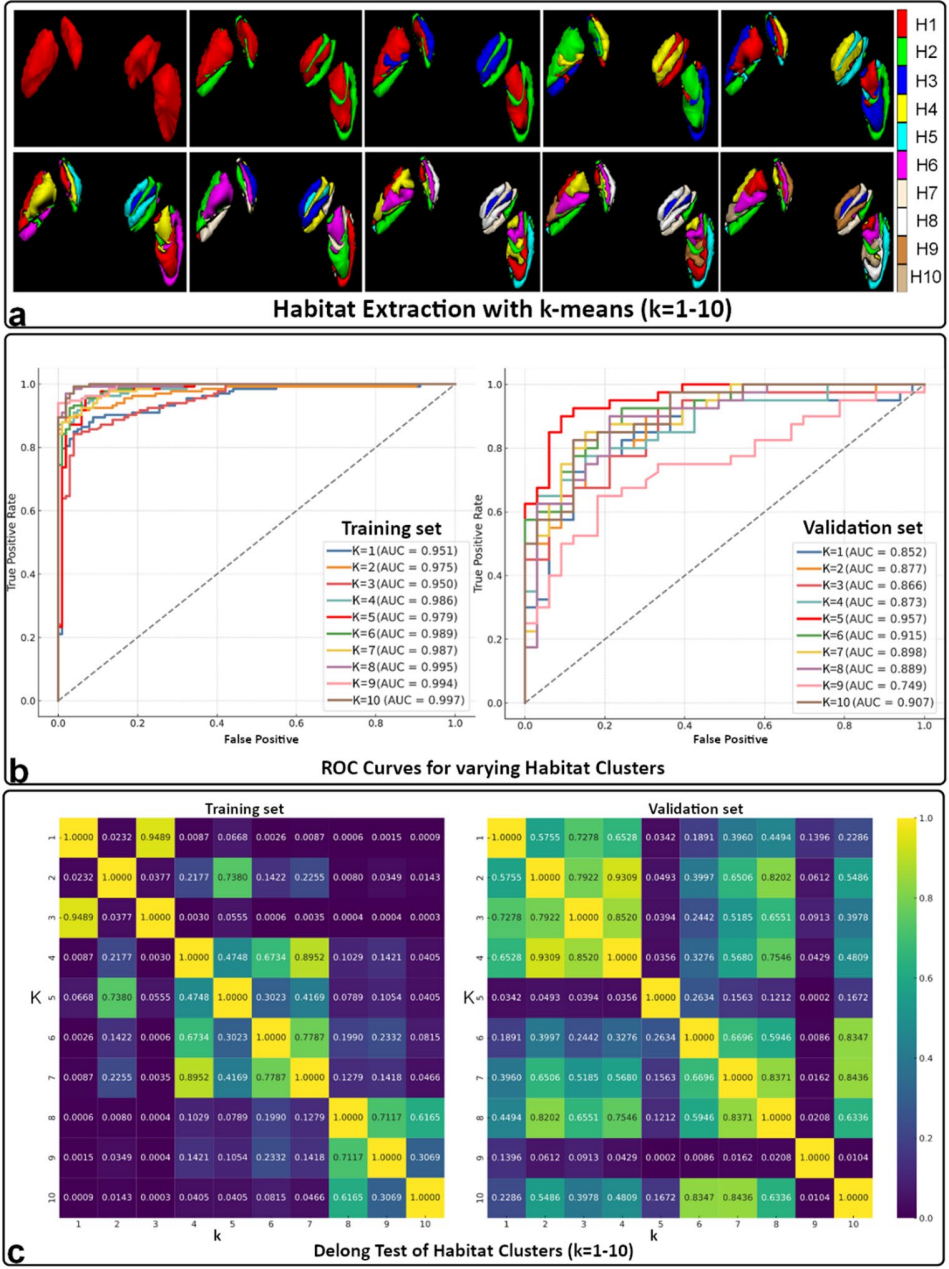
Analysis of Habitat-Based Radiomics and Model Evaluation. (**a**) Habitat Area Extraction: Visualization of habitat areas for k-means clusters ranging from 1 to 10, with different colors representing distinct habitats (H1 to H10**).** (**b**) ROC Curves for varying Habitat Clusters: ROC curves for clusters 1 to 10 on the training set (left) and external validation set (right), showing model performance with AUC values. (**c**) Delong Test of Habitat Clusters (k = 1–10): Heatmaps of p-values from the Delong test comparing ROC curves of different clusters on the training set (left) and external validation set (right), indicating statistical significance to identify the best cluster configuration.

This study utilized habitat-based radiomics to achieve improved PD diagnosis accuracy. VOIs were segmented into multiple sub-regions, or habitats, based on 19 unique features capturing intensity variations and textural patterns. K-means clustering, an unsupervised learning method that groups similar data points together, was used to identify these optimal habitats for accurate PD prediction. Figure 1a demonstrates the segmentation results (k-means clustering with k = 1–10) for habitat area extraction. Each distinct sub-region within the VOIs is visualized with a unique color (as shown in the color bar). Leveraging T1W and T2W image features, this study effectively captured spatial heterogeneity, emphasizing the critical role of detailed subregional segmentation for subsequent analysis. A multi-faceted approach yielded highly reliable and clinically applicable radiomics models. Optimized feature selection for each habitat was achieved by employing a comprehensive process involving Pearson correlation, minimum redundancy maximum relevance (mRMR), and least absolute shrinkage and selection operator (LASSO) regression. Subsequently, SVM models were developed to predict PD and elucidate the relationship between habitat features and clinical outcomes. Compared to previous PD research, our approach demonstrated significantly enhanced predictive capabilities.

### SVM performance with varying habitat clusters

Figure 1b shows the performance of our models using different numbers of habitat clusters (k = 1 to 10). It displays two sets of receiver operating characteristic (ROC) curves, one for the training set (left) and one for the external validation set (right). Ideally, the curve should hug the top left corner, indicating high accuracy with minimal false positives. Overall, the models performed very well, with AUC values ranging from 0.840 to 0.997. As expected, the training set showed slightly better performance than the validation set. For the training set, the AUC increased with more clusters, suggesting a benefit from detailed sub-region analysis. However, the validation set peaked at 5 clusters with an AUC of 0.957, which is significantly better than previously reported results^25,36,37^.

**Fig. 2:**
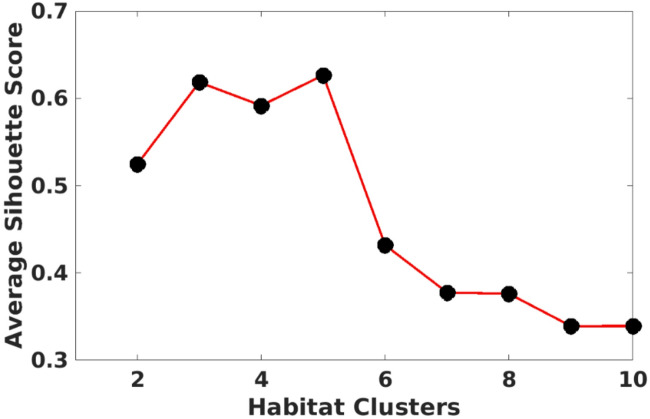
Average silhouette scores of SVM models with varying numbers of habitat clusters (k = 1–10) for the validation dataset. The silhouette score measures the quality of cluster assignments, with higher values indicating better separation between clusters.

Figure 1c shows the results of Delong tests, which statistically compare the performance of models with different habitat clusters. This confirms our observations: (1) In the training set, models with very few (k = 1,3) or many clusters (k = 8–10) performed similarly (either low or high AUC). Models with k = 4 and 6–7 showed statistically different performances. (2) In the validation set, the model with k = 5 clusters stood out with the highest AUC (0.957), significantly better than others. Table 1; Fig. 2 provide a more comprehensive picture of model performance with varying clusters (k = 1 to 10). These metrics include accuracy, sensitivity (correctly identified PD cases), specificity (correctly identified healthy controls), and 95% confidence intervals (CI). Overall, all models performed well, but there were variations. For the training set, accuracy and AUC steadily improved with increasing clusters. For the validation set, the model with 5 habitat clusters (k = 5) excelled, demonstrated the best performance with an AUC of 0.957 and accuracy of 0.89.

**Fig. 3:**
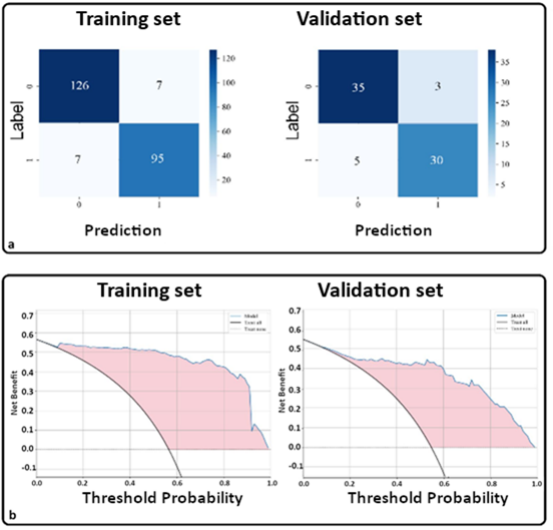
SVM Model Performance with 5 Habitat Clusters (k = 5). (**a**) Confusion Matrix: This table visualizes the model’s classification accuracy for both the training data (left) and external validation set (right). It breaks down the results based on true and predicted classes. Here’s an explanation of each cell: both the training data (left) and an independent validation set (right). It breaks down the results based on true and predicted classes. True positives (TP) represent correctly classified positive cases (top-left cell). Conversely, false positives (FP) indicate negative cases mistakenly classified as positive (top-right cell). Similarly, false negatives (FN) are positive cases incorrectly classified as negative (bottom-left cell), while true negatives (TN) are negative cases correctly identified (bottom-right cell). (**b**) Decision curve analysis (DCA): This analysis goes beyond raw accuracy by evaluating the model’s net benefit at different classification thresholds for both patient cohorts (left and right). The blue line represents the model’s net benefit, considering the benefits of correctly identifying positive cases (true positives) against the costs of incorrectly identifying negative ones (false positives). The black and dotted lines represent the net benefit of treating all or none of the patients, respectively. The shaded area highlights the region where the model offers a net benefit compared to these simpler strategies.

Figure 3a shows confusion matrices for the model with 5 habitat clusters, detailing its performance on both the training and validation sets. A confusion matrix summarizes how well the model classified patients. Ideally, most entries should lie on the diagonal (correct classifications). For the training set, the model misclassified 7 PD patients (out of 133) and 7 healthy controls (out of 102). Similarly, for the validation set, it misclassified 5 PD patients (out of 40) and 3 healthy controls (out of 33). These results translate to high true positive rates of 94.7% and 87.5% for training and validation sets, respectively. Figure 3b depicts the decision curve analysis (DCA) plots, which assess the clinical usefulness of a diagnostic test. Here, the DCA curves show the added benefit of using the model’s predicted probabilities for PD diagnosis compared to other approaches. The shaded area under the curve represents the net benefit, which is the potential gain in true positives minus the cost of false positives. Notably, the shaded area for the training set is larger than the validation set, reflecting a higher potential benefit for the training data. However, even for the validation set, the model offers meaningful improvements. For example, setting a probability threshold of 0.2 would reduce false positives by 29 cases per 100 patients in the training set and 8 cases per 100 patients in the validation set. These results suggest that the model can substantially improve clinical outcomes by aiding clinicians in identifying patients who would benefit from early intervention or further diagnostic testing, such as DaTscan, while minimizing unnecessary procedures for those unlikely to have PD. For instance, a threshold of 0.2 could be used to prioritize patients for confirmatory imaging, optimizing resource allocation in clinical settings.

Figure 2 sheds light on why 5 clusters might be ideal. This plot shows the average silhouette score, which measures how well data points are grouped within their clusters. A higher score indicates better separation between clusters. Here, 5 clusters achieved the highest score (0.6265), suggesting well-defined and distinct clusters. In a clinical context, this silhouette score indicates that the habitats identified within the caudate nucleus and putamen are meaningfully distinct, reflecting localized variations in tissue properties (e.g., texture or intensity) that are likely associated with PD-related neurodegeneration. This robust segmentation enhances the model’s ability to detect subtle, disease-specific changes, thereby improving diagnostic accuracy and supporting early identification of PD. Both performance metrics and silhouette scores consistently point to 5 clusters as the optimal choice for habitat segmentation and SVM model construction. This configuration effectively groups data points, allowing the model to capture relevant features and spatial variations within the brain regions. Consequently, the model with 5 habitat clusters offers superior accuracy, robustness, and generalizability for PD prediction.

### Optimal SVM model with 5 habitat clusters

Figure 3a shows confusion matrices for the model with 5 habitat clusters, detailing its performance on both the training and validation sets. A confusion matrix summarizes how well the model classified patients. Ideally, most entries should lie on the diagonal (correct classifications). For the training set, the model misclassified 7 PD patients (out of 133) and 7 healthy controls (out of 102). Similarly, for the validation set, it misclassified 5 PD patients (out of 40) and 3 healthy controls (out of 33). These results translate to high true positive rates of 94.7% and 87.5% for training and validation sets, respectively. Figure 3b depicts the decision curve analysis (DCA) plots, which assess the clinical usefulness of a diagnostic test. Here, the DCA curves show the added benefit of using the model’s predicted probabilities for PD diagnosis compared to other approaches. The shaded area under the curve represents the net benefit, which is the potential gain in true positives minus the cost of false positives. Notably, the shaded area for the training set is larger than the validation set, reflecting a higher potential benefit for the training data. However, even for the validation set, the model offers meaningful improvements. For example, setting a probability threshold of 0.2 would reduce false positives by 29 cases per 100 patients in the training set and 8 cases per 100 patients in the validation set. These results, combined with the high accuracy metrics, suggest the model can substantially improve clinical outcomes.

**Table 1:** Performance metrics for SVM models with varying habitat clusters (k = 1–10).

k	Accuracy	AUC	95% CI	Sensitivity	Specificity	Dataset
1	0.894	0.951	0.924–0.978	0.842	0.961	Train
	0.781	0.852	0.761–0.944	0.700	0.879	Validation
2	0.928	0.975	0.957–0.993	0.887	0.980	Train
	0.781	0.877	0.800–0.955	0.725	0.848	Validation
3	0.889	0.950	0.925–0.976	0.835	0.961	Train
	0.795	0.866	0.783–0.949	0.900	0.667	Validation
4	0.936	0.986	0.976–0.996	0.955	0.912	Train
	0.795	0.873	0.794–0.953	0.750	0.848	Validation
5	0.940	0.979	0.961–0.997	0.947	0.931	Train
	0.890	0.957	0.917–0.997	0.875	0.909	Validation
6	0.945	0.989	0.980–0.997	0.947	0.941	Train
	0.836	0.915	0.854–0.976	0.900	0.758	Validation
7	0.923	0.987	0.978–0.996	0.872	0.990	Train
	0.836	0.898	0.827–0.970	0.825	0.848	Validation
8	0.970	0.995	0.990–1.000	0.962	0.980	Train
	0.836	0.889	0.813–0.965	0.875	0.788	Validation
9	0.962	0.994	0.988–0.999	0.932	1.000	Train
	0.712	0.749	0.636–0.862	0.625	0.818	Validation
10	0.974	0.997	0.993–1.000	0.985	0.961	Train
	0.836	0.907	0.842–0.972	0.800	0.879	Validation

### SHAP analysis of the SVM model with 5 habitat clusters

To gain deeper insights into the workings of our SVM model with 5 habitat clusters and enhance trust in its predictions, we conducted a SHAP (SHapley Additive exPlanations) analysis. SHAP assigns importance values to each feature, revealing their contribution to differentiating HC from PD patients.

**Fig. 4:**
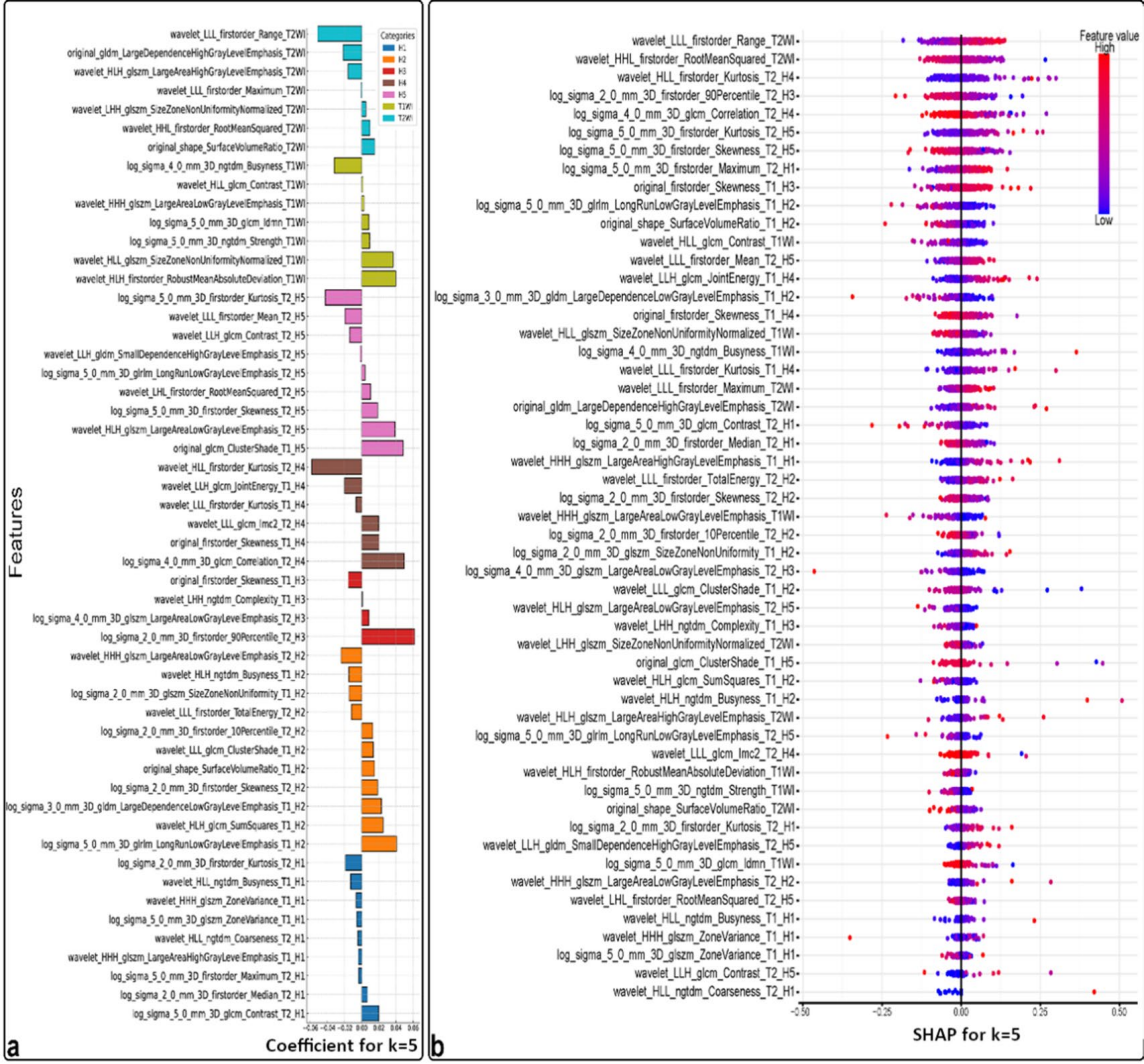
Feature Importance Analysis for SVM Model with 5 Habitat Clusters (k = 5). (**a**) Feature Coefficients: This plot shows the relative importance of each selected feature, categorized by habitat type (H1-H5) and imaging contrast (T1WI, T2WI). Higher coefficient values indicate greater influence on the model’s predictions. (**b**) SHAP Bee Swarm Plot: This plot visualizes the impact of individual features on model predictions for specific data points. Each facet represents a feature, with color intensity indicating its influence on the prediction. The position of each data point along the x-axis reflects its overall feature value. By observing how data points cluster within facets, we can understand how different feature values interact to influence model predictions.

Figure 4a presents the feature importance analysis, encompassing 52 features across 12 categories (T1W, T2W, and H1-5). SHAP successfully identified key features, primarily from T2W contrast, that effectively distinguish HC from PD patients by capturing significant structural and textural brain variations. Figure 4b employs a SHAP bee swarm plot to visualize how individual features influence predictions for each data point. Analyzing the distribution of “bees” within each feature facet provides insights into feature interactions and their impact on predictions.

**Fig. 5:**
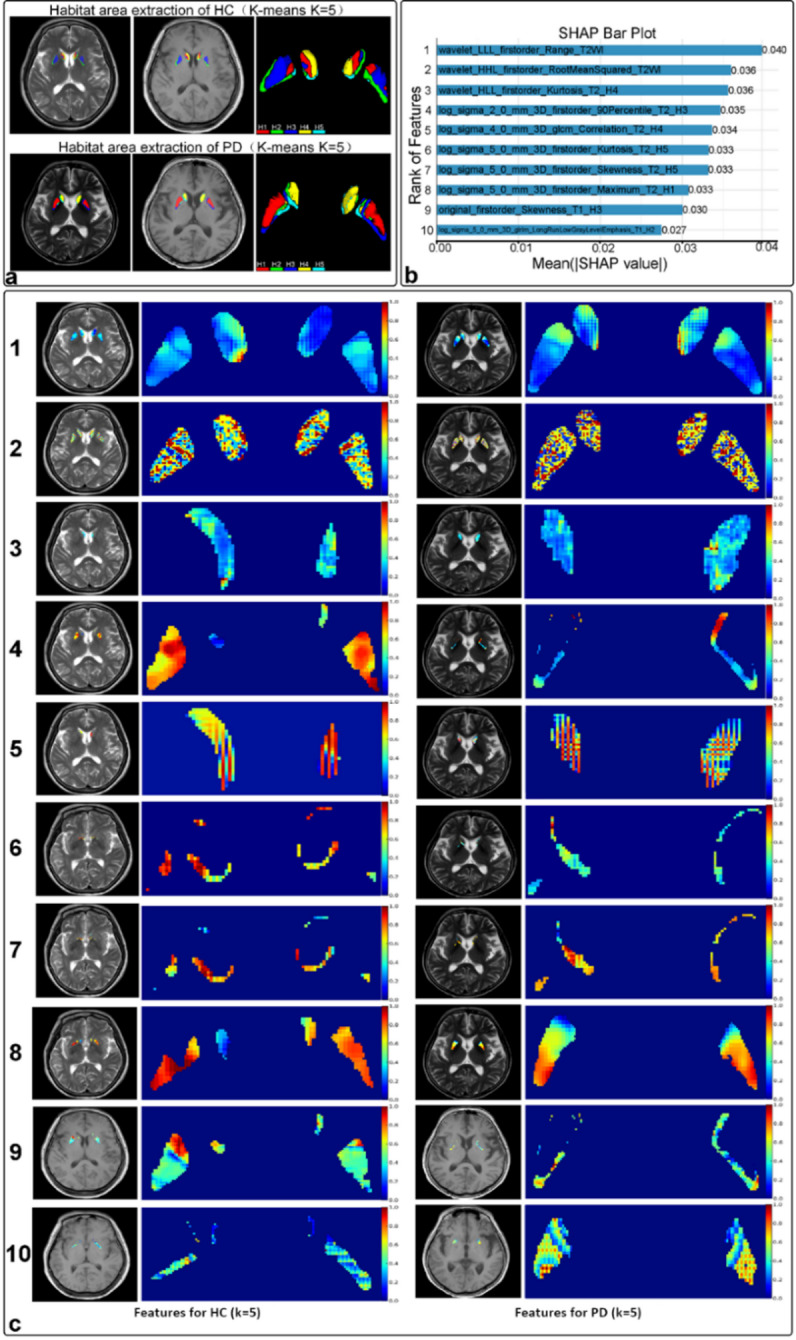
Top 10 Features for SVM Model with 5 Habitat Clusters. (**a**) Habitat Segmentation: Displays of the VOI segmentation into 5 habitat clusters for both HC (upper panel) and PD patients (lower panel). (**b**) Top 10 Radiomic Features: The SHAP bar plot shows the average SHAP value for the top 10 most important radiomic features, ranked in ascending order of importance. Higher SHAP values indicate a greater influence on the model’s predictions. (**c**) Spatial Distribution of Top Features: This panel visualizes the spatial distribution of the top 10 radiomic features ranked by their SHAP importance. By comparing HC and PD patients, we can identify distinct patterns and potential differences.

Focusing on the top 10 most impactful features, Fig. 5a highlights their contributions. Figure 5b presents SHAP bar plots illustrating the average SHAP value for these features, ranging from 0.04 to 0.027, and accounting for up to 32.7% of the predictive power. Figure 5c visualizes the spatial distribution of these top features within brain regions for both HC and PD subjects, emphasizing their predominance in T2W contrast images. These top 10 features primarily reflect changes in intensity range, intensity RMS, and kurtosis within habitats such as the H4 region in the caudate nucleus and H3 and H5 in the putamen. As summarized in Table 2; Fig. 5c clearly demonstrates differences between HC and PD subjects. HC subjects exhibit more uniform and lower intensity values, while PD patients show increased heterogeneity, higher intensity variations, and greater skewness in intensity distribution.

**Table 2:** Top 10 most important features for the SVM model with 5 habitat clusters (k = 5).

Rank	Feature	SHAP	HC characteristics	PD Characteristics
1	wavelet_LLL_1storder_ Range_T2W	0.040	homogeneous, lower intensity values, less variability	higher feature values, greater variability, uneven distribution
2	wavelet_HHL_1storder_ RMS_T2W	0.036	more uniformity, fewer extreme values (red & blue)	greater tissue heterogeneity with more extreme values
3	wavelet_HLL_1storder_ Kurtosis_T2_H4	0.036	consistent kurtosis, less variation and a slender shape	higher variations with a fuller and more central shape.
4	log_sigma_2mm_3D_1st order_90Percentile_T2_H3	0.035	within putamen, more high intensity values	primarily at putamen boundary with lower intensity values
5	log_sigma_4mm_3D_glcm_Correlation_T2_H3	0.034	uniform correlations with consistent texture patterns	reduced uniformity, enhanced complexity, higher central values
6	log_sigma_5mm_3D_1st order_Kurtosis_T2_H5	0.033	Feature values are mostly high (close to red)	predominantly low values (leaning towards blue)
7	log_sigma_5mm_3D_1st order_Skewness_T2_H5	0.033	closer to enclosed edge boundaries in shape	similar intensity but significant different in shape
8	log_sigma_5mm_3D_1st order_Maximum_T2_H1	0.033	high feature values both putamen and caudate nucleus	confined to putamen with a gradient from low to high values
9	Original_1storder_ Skewness_T1_H3	0.030	mainly within putamen with more high-value regions	At putamen’s boundary areas with less high-value regions
10	log_sigma_5mm_3D_ glrlm_LongRunLow_Gray Level_T1_H2	0.027	elongated shape and primarily low values	clumped with many medium to high feature values

To assess the stability of these top 10 features, we analyzed their selection frequency across the ten-fold cross-validation folds used in the training process. All top 10 features were selected in at least 8 out of 10 folds (80% frequency), with the top three features (wavelet_LLL_1storder_Range_T2W, wavelet_HHL_1storder_RMS_T2W, and wavelet_HLL_1storder_Kurtosis_T2_H4) appearing in all 10 folds (100% frequency). This high selection consistency indicates that these features are robust across different data splits. Additionally, we calculated the intraclass correlation coefficient (ICC) for these features to evaluate their reliability across cross-validation folds and across a subset of 20 scans (10 from each center, balanced for PD and HC) acquired on different scanners (1.5T at Li-Cheng, 3.0T at Dong-Hai). The ICC values for the top 10 features ranged from 0.76 to 0.89 (mean ICC = 0.82), indicating good to excellent reliability. These results suggest that the features are stable despite scanner variability, likely due to the preprocessing with N4 Bias Field Correction and the rigorous feature selection process (Pearson correlation, mRMR, LASSO regression), which prioritized features less sensitive to acquisition differences. These findings suggest significant structural and textural alterations in the brains of PD patients compared to HC subjects. The combination of habitat-based radiomics and SHAP analysis proves effective in capturing disease-related changes, leading to improved diagnostic accuracy. By understanding the impact of key features through SHAP analysis, we gain valuable insights into PD pathology and enhance the interpretability of model prediction.

## Discussion

This study investigated the potential of a novel method called habitat-based radiomics for diagnosing PD using routine MRI scans. Here are the key findings: (1) Habitat-based radiomics achieved high accuracy in PD diagnosis. The method segmented brain regions (caudate nucleus and putamen) into sub-regions (habitats) based on MRI features. This segmentation improved the accuracy of a machine learning model in differentiating PD patients from HC compared to traditional methods. (2) The model using 5 habitat clusters achieved the best results. The model using 5 clusters performed best, achieving over 94% accuracy in identifying PD and significantly reducing false positives. The silhouette score of 0.6265 for the 5-cluster configuration indicates moderate to good clustering quality, suggesting that the identified habitats represent distinct sub-regions with unique imaging characteristics. Clinically, this implies that our model can detect localized pathological changes, such as neuronal loss or microstructural alterations, which are critical for early PD diagnosis. This segmentation enhances the model’s sensitivity to subtle disease markers, potentially allowing clinicians to identify PD at an earlier stage when interventions are most effective.(3) SHAP analysis revealed key features for PD detection. The analysis identified features from T2W images that captured structural and textural changes in brain regions associated with PD, suggesting that these features are crucial for differentiating PD patients from HC. Specifically, features such as increased intensity range, intensity root mean square (RMS), kurtosis, and skewness in habitats within the caudate nucleus and putamen reflect pathological changes in PD. These variations likely correspond to known neuropathological processes, including dopaminergic neuronal loss, gliosis, and iron accumulation in the basal ganglia. For instance, increased intensity variability and kurtosis in T2W images may indicate disrupted tissue homogeneity due to neuronal degeneration and microglial activation, which are prominent in PD-affected regions like the putamen. Similarly, higher skewness could reflect asymmetric intensity distributions caused by focal iron deposition, a known contributor to T2 signal changes in PD. The stability of these features, demonstrated by their high selection frequency (80–100%) across cross-validation folds and good to excellent ICC values (0.76–0.89), underscores their robustness and reliability, even across different MRI scanners. This stability enhances confidence in the model’s applicability in diverse clinical settings, where scanner variability is common, and supports the potential for these features to serve as reliable biomarkers for PD diagnosis. These radiomic features provide a quantitative link to microstructural alterations, enhancing the biological interpretability of our model and its ability to detect early PD pathology. Overall, the study demonstrates that habitat-based radiomics is a promising approach for diagnosing PD using routine MRI scans. It offers improved accuracy compared to traditional methods and provides valuable insights into the underlying disease processes.

The DCA results further underscore the clinical utility of our model. By quantifying the net benefit across various probability thresholds, the DCA demonstrates that our model can improve clinical decision-making by balancing the benefits of correctly identifying PD cases against the costs of false positives. For example, at a probability threshold of 0.2, the model reduces false positives by 8 cases per 100 patients in the validation set, which could translate to fewer unnecessary diagnostic tests or treatments in clinical practice. In a practical scenario, clinicians could use this threshold to identify patients who require confirmatory imaging (e.g., DaTscan) or early therapeutic intervention, such as initiating neuroprotective treatments. Alternatively, a higher threshold (e.g., 0.5) could be used to confidently rule out PD in low-risk patients, avoiding costly and invasive procedures. This flexibility allows the model to be tailored to specific clinical needs, such as prioritizing early detection in high-risk populations or minimizing overdiagnosis in screening settings. These decision-making scenarios highlight the model’s potential to optimize patient management and improve resource allocation in clinical settings.

The model is designed to serve as a decision support tool for neurologists and general radiologists in routine clinical practice. It can supplement DaTscan referrals by providing an initial, non-invasive assessment using widely available MRI scans, potentially reducing the need for more expensive or radiation-involving procedures. For instance, in scenarios where DaTscan is unavailable or contraindicated, or in resource-limited settings, MRI-based habitat-based radiomics offers a viable alternative for early PD screening. Target users include neurologists evaluating motor symptoms and radiologists interpreting brain scans, particularly in outpatient clinics or during initial diagnostic workups. The model’s interpretability, via SHAP analysis, allows clinicians to understand key features driving predictions, facilitating integration into multidisciplinary care teams.

For detailed MRI analysis with habitat-based radiomics, we used k-means clustering to construct the brain VOIs into different habitats. By analyzing these sub-regions in detail—extracting 1197 features from each—we captured variations within brain regions important for PD diagnosis. This approach provides a more nuanced picture of brain changes compared to traditional methods that only consider overall volume or structure. We tested ten different ways to divide the relevant anatomic VOIs into zones to find the most effective approach. The model with 5 habitat clusters provided the best results in all metrics (accuracy, AUC, sensitivity, specificity, and silhouette score). This highlights the value of habitat-based radiomics in capturing relevant markers of PD in routine clinical MRI scans. The different brain zones revealed distinct characteristics associated with PD, providing insights into the physical changes happening in the brain during the disease. Interestingly, features from different MRI contrasts (T1W and T2W) provided complementary information, underscoring the importance of a multi-faceted approach for PD diagnosis^25,37,38^.

### Comparison with traditional radiomic approaches

In comparison with traditional methods, habitat-based radiomics uses advanced techniques to analyze structural complexities, providing a more detailed assessment, while traditional methods often miss the subtle and varied nature of brain changes in PD. Traditional radiomics extracts features from the entire image or outlined VOI, treating it as a single unit, which can miss variations within different regions of the image^21,35^. In contrast, habitat-based radiomics addresses this limitation by segmenting the image into distinct functional or anatomical sub-regions (habitats) before feature extraction^29–33^. This allows it to capture the spatial heterogeneity within a brain region, crucial for diseases like Parkinson’s where specific areas are affected. For example, prior studies using traditional radiomics for PD diagnosis have reported moderate performance, with AUC values ranging from 0.80 to 0.87 and accuracies around 80–85% when analyzing whole-brain or basal ganglia regions using structural MRI or diffusion tensor imaging^26,27,35^. In contrast, our habitat-based radiomics model achieved an AUC of 0.957 and an accuracy of 0.89 in the external validation cohort, representing a significant improvement over these traditional approaches. This enhancement is likely due to the ability of habitat-based radiomics to detect localized pathological changes, such as textural variations in the caudate nucleus and putamen, which are averaged out in traditional radiomic analyses. By linking these textural variations to PD pathophysiology, such as neuronal loss and iron deposition, our approach not only improves diagnostic performance but also provides a deeper understanding of disease-related changes in the basal ganglia, aligning radiomic features with biological mechanisms.

The different brain zones revealed distinct characteristics associated with PD, providing insights into the physical changes happening in the brain during the disease prorgrssion. Sub-region segmentation within habitat-based radiomics presents a significant challenge due to the inherent heterogeneity in regions of interest. To effectively characterize these sub-regions and inform downstream analyses, identifying meaningful features is crucial. Unfortunately, a standardized approach for feature selection in this context is lacking, as optimal feature sets are highly dependent on imaging modality, disease type, and sub-region definitions. Given these complexities, this study leverages textual and intensity-based features, carefully considering their relevance, redundancy, and robustness for habitat segmentation. Interestingly, features from different MRI contrasts provided complementary information, underscoring the importance of a multi-faceted approach for PD diagnosis^17,37–39^. By capturing spatial variations, habitat-based radiomics can identify features specific to disease processes occurring in certain brain regions, leading to a more targeted and potentially more accurate diagnosis. Knowing the origin of features from specific habitats within the image helps in understanding the biological underpinnings of the disease, offering valuable insights for treatment development. Moreover, habitat-based analysis might allow for tailoring treatment plans based on the specific pattern of disease involvement within different brain regions.

Compared to other advanced imaging techniques, habitat-based radiomics also demonstrates competitive performance. For instance, multimodal PET/MRI radiomics approaches for distinguishing PD from atypical parkinsonism have achieved AUCs of 0.85–0.90, but these methods rely on costly and less accessible imaging modalities^36^. Similarly, DTI-based radiomics studies have reported AUCs of 0.82–0.88 for PD diagnosis, with accuracies typically below 85%, limited by the need for specialized imaging sequences^28,37^. In contrast, our approach leverages routine clinical MRI scans, achieving a higher AUC (0.957) and accuracy (0.89) while maintaining accessibility and scalability. This improvement highlights the advantage of habitat-based radiomics in extracting nuanced, spatially heterogeneous features from widely available T1W and T2W MRI data, reducing dependence on advanced imaging infrastructure. Furthermore, unlike studies combining imaging with cerebrospinal fluid (CSF) biomarkers, which may achieve AUCs up to 0.92 but require invasive procedures^40,41^, our method is non-invasive, enhancing its practicality for routine clinical use.

The landscape of PD diagnosis is undergoing rapid transformation, with a primary focus on earlier detection and heightened accuracy. Wearable technology, incorporating sensors like accelerometers and gyroscopes, is gaining prominence for monitoring PD symptoms, offering potential for early identification of both motor and non-motor manifestations^42^. Simultaneously, the pursuit of novel biomarkers, such as blood-based or imaging markers, coupled with advancements in imaging techniques like fMRI^23^, DTI^37^, and PET^43^, is enhancing diagnostic precision. In comparison with other advanced techniques, habitat-based radiomics, when combined with machine learning algorithms like SVMs, as used in this study, leverages the power of machine learning to identify complex patterns within extracted features, enhancing diagnosis and prediction. This technique can be applied to data from various imaging modalities. By analyzing features from each modality within specific habitats, it provides a more comprehensive picture of the disease and offers a more interpretable approach^25^. This allows researchers to understand which features from specific brain regions contribute most to the model’s predictions. Unlike other studies that rely on multiple data sources such as PET^43^ and CSF^40,41^ biomarkers, our method only relies on routine MRI scans, making it more accessible and scalable for clinical use. This data efficiency enhances its practicality in clinical settings. Overall, habitat-based radiomics provides a valuable method for analyzing medical images by capturing spatial variations within tissues. It offers advantages in disease specificity, interpretability, and potential for personalized medicine, making it a promising tool for clinical applications. Future research should explore combining habitat-based radiomics with other data sources to potentially improve diagnostic accuracy further.

### Our study has limitations

The limited sample size, particularly the validation cohort (*n* = 73), necessitates further validation of our findings using larger datasets. While this sample size was sufficient for establishing proof-of-concept, larger validation studies (e.g., *n* > 200 per site) are required to confirm clinical utility and generalizability across diverse populations and settings. To enhance the robustness of our results, using larger datasets with incorporation of deep learning techniques holds promise. Additionally, manual segmentation of the caudate nucleus and putamen, while performed by experienced radiologists, is susceptible to inter-rater variability, which could affect the reproducibility of our findings. To minimize this variability, we employed a standardized segmentation protocol and ensured that segmentations were conducted by radiologists with over 15 years of brain MRI experience, who were blinded to patient diagnoses to reduce bias. Furthermore, we conducted a preliminary inter-rater reliability assessment using a subset of 30 MRI scans, achieving an intraclass correlation coefficient of 0.82 for the delineated volumes of interest (VOIs), indicating good agreement among raters. Despite these efforts, manual segmentation remains a limitation, particularly given the time-intensive nature of the process and the potential for subtle differences in delineations. Automated segmentation algorithms could improve consistency and efficiency, but our experiments with such methods on rapid clinical MRI protocols with relatively thick slices (6 mm) showed insufficient accuracy for reliable application in this study. Future work should focus on developing or adapting automated segmentation tools optimized for clinical MRI data to enhance reproducibility and scalability. Additionally, manual segmentation of the caudate nuclei and putamen is susceptible to inter-rater variability, highlighting the potential benefits of automated segmentation algorithms for improved consistency and reliability^14,15^. However, the rapid clinical MRI protocols with relatively thick slices employed in this study pose challenges for automatic segmentation methods, as our experiments indicate insufficient accuracy for enhancing reproducibility with such data.

The SVM model with 5 habitat clusters likely strikes a balance between capturing detailed features and maintaining generalizability. It extracts a comprehensive feature set from relevant brain regions without overfitting to the training data. This balance enables good performance on unseen data in the validation set. However, some performance fluctuations observed between training and validation sets with increasing cluster numbers could be due to random variations within each cluster or limitations of the SVM model itself, particularly with a limited dataset^44^. SVM models are susceptible to overfitting, especially when dealing with a large number of features at higher cluster numbers and a small dataset. Exploring alternative machine learning models less prone to overfitting, such as Random Forests^45^ or XGBoost^46^, could be beneficial for future studies.

No additional harmonization techniques, such as ComBat, were applied, as our study aimed to develop a model robust to real-world clinical variability, where MRI scans are often acquired using diverse equipment and protocols. This decision highlights the model’s potential for broad clinical applicability but also underscores the need for further testing in even more heterogeneous settings to confirm its generalizability. While our current study provides promising results, we recognize that further research is needed to fully establish the predictive value of habitat-based radiomics for PD diagnosis. To assess the method’s ability to predict disease progression over time, we have conducted additional analyses of open-source MRI data with multiple measurements over time (https://www.ppmi-info.org/access-data-specimens/download-data) using survival analysis techniques. Initial findings demonstrate a significant association between habitat-based radiomic features and disease progression, suggesting that our approach may have potential as a prognostic biomarker. While further research is needed to fully validate its predictive capabilities, our results provide a promising foundation for future studies.

The complexity of our proposed method can be a potential concern for clinical applications. We are exploring the use of more computationally efficient machine learning algorithms to reduce the computational burden. This will make our method more practical for clinical implementation. However, we acknowledge that further research is needed to fully evaluate the impact of these simplifications on the method’s accuracy and generalizability.

*In conclusion*, habitat-based radiomics analysis of routine clinical MRI data demonstrates significant potential for enhancing PD diagnosis. Novel in its approach, this method leverages spatial variations within the caudate and putamen to extract more nuanced information than traditional techniques. Our findings underscore the efficacy of this approach, with an SVM model achieving an AUC of 0.957 and accuracy of 0.89 in external validation. By linking radiomic features to PD pathophysiology, our study not only enhances diagnostic accuracy but also provides insights into the biological mechanisms underlying basal ganglia degeneration, paving the way for more targeted diagnostic and therapeutic strategies. The stability and reliability of the top radiomic features, as evidenced by their consistent selection and high ICC values across diverse scanner settings, further support the robustness of our approach, making it a promising tool for clinical translation. By utilizing readily available MRI scans and offering disease specificity, habitat-based radiomics presents several advantages. While limitations such as sample size and manual segmentation persist, our efforts to minimize inter-rater variability through standardized protocols and reliability assessments provide a foundation for robust segmentation, though future adoption of automated methods could further enhance reproducibility. This study highlights its promise as a powerful tool for accurate PD detection and improved clinical outcomes. Future research with larger datasets and automated segmentation methods is warranted to solidify the clinical utility of this approach.

## Methods

### Study subjects and the inclusion criteria

This study enrolled a total of 308 participants (173 PD, 135 HC) across two medical centers at Fujian Medical University: Li-Cheng (235 participants; 133 PD, 102 HC) and Dong-Hai (73 participants; 40 PD, 33 HC).

All experimental procedures involving human subjects were conducted in accordance with the Declaration of Helsinki and approved by the local institutional review committee (IRB) at the 2nd Affiliated Hospital of Fujian Medical University (IRB protocol number: 2024:787). Informed consent was obtained from all participants prior to their inclusion in the study.

For PD patients, inclusion criteria included a clinically confirmed diagnosis based on the Movement Disorder Society Clinical Diagnostic Criteria for Parkinson’s Disease (Postuma et al., 2015), age between 50 and 80 years, disease duration under 10 years, ability to undergo MRI scans, and no severe cognitive impairment (Mini-Mental State Examination score ≥ 24). No formal staging (e.g., Hoehn and Yahr scale) or further stratifications (e.g., by motor subtypes such as tremor-dominant or akinetic-rigid, or by non-motor features) were applied; patients were analyzed as a single PD cohort to focus on general diagnostic differentiation from healthy controls. Additionally, patients with atypical or secondary parkinsonism, severe brain function-altering comorbidities, prior brain surgery for PD, or MRI contraindications were excluded. HC subjects were age- and sex-matched to the PD group, with no history of neurological or psychiatric disorders and no MRI contraindications. This approach ensured a comparable and well-defined study population (Table 3).

Table 3 presents the median age (with standard deviation) and sex distribution (counts and proportions) for PD and HC groups within both the training cohort and the external validation cohort. The p-values indicate the results of Pearson’s chi-squared tests comparing the differences between PD and HC groups within each cohort, as well as between the overall training and validation cohorts. The results suggest successful group matching, with no significant differences in age or sex distribution between PD and HC groups across both cohorts, nor between the training and external validation cohorts.

By adhering to these inclusion and exclusion criteria, the study ensured a well-defined and consistent patient cohort for investigating the application of habitat-based radiomics in Parkinson’s disease. Utilizing the two centers for training and external validation cohorts enhances the study’s robustness and generalizability.

### MRI scanning parameters

In this study, MRI scans were conducted using different MRI equipment, including T1-weighted (T1W) and T2-weighted (T2W) imaging contrasts. The Li-Cheng center (training cohort) utilized Philips and GE Medical 1.5T MRI machines, while the Donghai center (external validation cohort) used a Philips 3.0T MRI machine.

For the Li-Cheng center, the T1W acquisition parameters were as follows: axial plane, slice thickness of 6 mm, interslice gap of 2 mm, repetition time (TR) of 2250ms, echo time (TE) of 24ms, field of view (FOV) of 240 mm ×240 mm, matrix size of 512 × 512, and 1 signal averages. The T2WI parameters included a TR of 8500ms, TE of 155ms, with the same FOV, matrix size, slice thickness, and interslice gap as T1W.

At the Dong-Hai center, the T1W acquisition parameters were axial plane, slice thickness of 6 mm, interslice gap of 2 mm, TR of 2500ms, TE of 23ms, FOV of 240 mm × 240 mm, matrix size of 512 × 512, and 1 signal average. For T2W acquisition, the parameters were a TR of 4000ms, TE of 104ms, with the same FOV, matrix size, slice thickness, NEX and interslice gap as T1W. To ensure comparability of radiomic features extracted from scans acquired on different MRI scanners, all images underwent preprocessing to correct for intensity inhomogeneities using the N4 Bias Field Correction algorithm^47^. This step mitigated variations in signal intensity that could arise from differences in scanner hardware or imaging parameters, enhancing the consistency of feature extraction across datasets. No additional harmonization techniques, such as ComBat, were applied, as our study aimed to develop a model robust to real-world clinical variability, where MRI scans are often acquired using diverse equipment and protocols (see “VOI Delineation and Sub-Region Clustering” subsection for further details). The inclusion of data from different scanners strengthens the generalizability of our findings, as it reflects the heterogeneity encountered in clinical practice.

Utilizing different MRI scanners and settings across the training and validation cohorts strengthens the study’s generalizability. By incorporating data acquired on various machines, the developed models are more likely to perform well in real-world clinical settings with diverse MRI equipment.

**Table 3:** Demographic of the training and external validation Cohorts.

Parameter	Training cohort(*n* = 235)			External validation cohort(*n* = 73)					*P*
	PD (*n* = 133)	HC (*n* = 102)	*P*	PD (*n* = 40)		HC (*n* = 33)		*P*	
Age									
Median (SD)	69(9.05)	67(6.13)	0.587	67(10.65)		65(4.05)		0.903	0.338
	68(7.94)			65(8.29)					
Chi-squared	4.543								
Sex									
Female	128(54.47%)			36(49.32%)					0.524
	65(48.87%)	63(61.76%)	0.067	18(45.00)	18(54.55)		0.564		
Male	68(51.13%)	39(38.24%)		22(55.00)	15(45.45)				
	107(45.53%)			37(50.68%)					
Chi-squared	0.405								

*P* – value was calculated using Pearson’s Chi-square test.

### VOI delineation and sub-region clustering

**Fig. 6:**
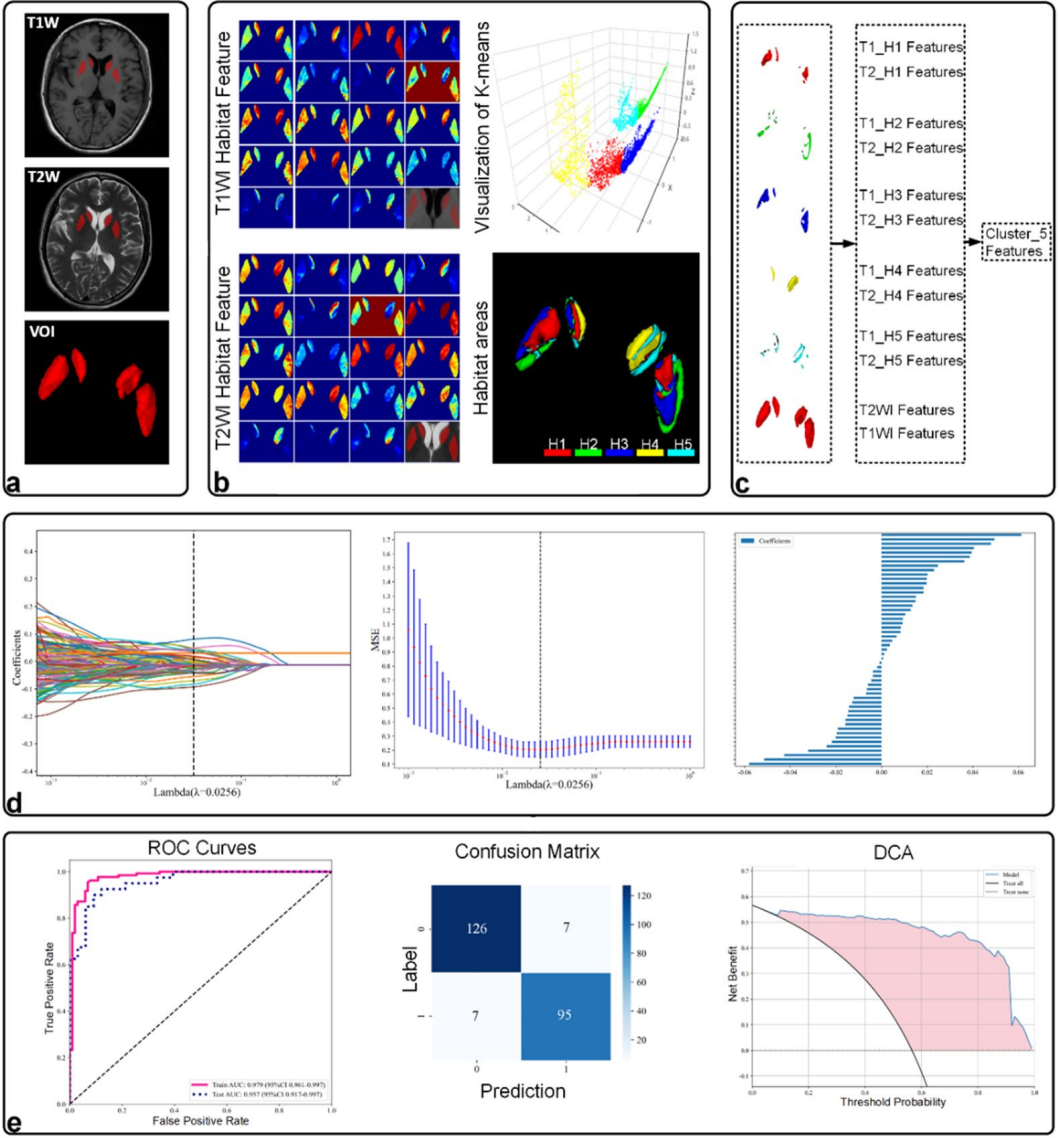
The workflow for Habitat-based radiomics analysis and model development procedures. (**a**) VOI Segmentation: T1W and T2W images were used to delineate VOIs, focusing on key brain structures related to PD. (**b**) Habitat Area Generation: Using k-means clustering, different habitat areas were generated based on the voxel radiomic features extracted from T1W and T2W images. (**c**) Radiomics Feature Extraction: a comprehensive set of radiomic features was extracted for each habitat area and the overall ROIs from both T1W and T2W images. (**d**) Feature Selection: The feature selection process included three steps: filtering based on the Pearson correlation coefficient, mRMR, and LASSO regression to refine the feature set. (**e**) Model Validation: The selected features were used to train SVM models. Evaluation metrics include AUC, confusion matrix, and DCA curves to assess the model’s predictive accuracy, sensitivity, specificity, and clinical utility.

We delineated the VOIs within the scans. This involved manually tracing the caudate nucleus and putamen on both T1W and T2W images using ITK-SNAP software (version 4.0)^48^. Experienced radiologists (over 15 years of brain MRI experience) performed this segmentation independently, blinded to patient diagnoses. This meticulous approach minimized bias and ensured accurate representation of anatomical structures crucial for subsequent analysis (Fig. 6a).

We then employed habitat-based radiomics to analyze these VOIs. This technique segments the delineated VOIs into sub-regions (habitats) based on specific intensity and texture variations within the MRI data. For each MRI contrast (T1W and T2W), we extracted 19 features from each voxel within a VOI. These features captured various aspects of the image data, including: (1) First-order statistics (e.g., entropy, mean absolute deviation); (2) Gray-level co-occurrence matrix^49^ (GLCM) features; (3) Gray-level run-length matrix^50^ (GLRLM) features; (4) Gray-level size zone matrix (GLSZM) features; (5) Neighborhood gray-tone difference matrix (NGTDM) features^51^. To further enhance the robustness of feature extraction across different scanners, our feature selection process (described in the Feature Extraction and Selection subsection) prioritized features that were stable and less sensitive to scanner-related variations. Specifically, the use of Pearson correlation, minimum redundancy maximum relevance (mRMR), and LASSO regression ensured that only the most relevant and non-redundant features were retained, mitigating potential biases introduced by scanner heterogeneity. This approach, combined with the high performance of our model in the external validation cohort (AUC of 0.957), suggests that our radiomic features were robust to variations in MRI acquisition.

Table 4 summarizes these habitat features with their corresponding definitions. By analyzing these features, we were able to generate distinct sub-regions (habitats) within the VOIs. This approach provided a detailed characterization of the spatial heterogeneity and texture variations known to be associated with PD abnormalities. We typically achieved this using k-means clustering^52^, which classifies voxels into separate habitats based on their extracted features (Fig. 6b). To identify the optimal segmentation for model performance we tested a range of habitat clusters (k) from 1 to 10. Figure 6b illustrates the clustering results using a k-means algorithm with 5 habitat clusters (k = 5).

Furthermore, we developed ten different models by incorporating varying numbers of habitat clusters (k = 1–10) to assess their effectiveness in predicting PD. This systematic evaluation allowed us to determine the optimal model configuration that yielded the highest predictive accuracy. By analyzing how VOI segmentation into different habitats affects model performance, we aimed to enhance the overall predictive power and clinical utility of these models for PD research.

### Feature extraction and selection

As illustrated in Fig. 6c, we extracted a large number of radiomic features from each segmented habitat area and the overall VOI. For each habitat area and the overall ROI, 1,197 features were extracted based on both T1W and T2W data^35^. For example, with 5 habitat clusters (k = 5), we extracted features for T1-Habitats 1 to 5 (T1-H1 to T1-H5), T2-Habitats 1 to 5 (T2-H1 to T2-H5), and the entire T1W and T2W VOIs, resulting in a total of 12 categories. Each of these 12 categories comprised 1,197 features, including 234 First-Order Statistical features, 286 GLCM features, 182 GLDM features, 208 GLRLM features, 208 GLSZM features, 65 NGTDM features, and 14 Shape features, resulting in a total of 14,364 features for the 5-cluster configuration. We conducted systematic studies for model configurations with habitat clusters ranging from 1 to 10 (k = 1–10), and the total number of extracted features for each model is therefore equal to 1,197 × (2k + 2).

**Table 4:** Features used for habitat segmentation.

Feature	Description
First-Order Statistical Features	
Original_1storder_Entropy	Randomness and complexity of gray level distribution
original_1storder_MeanAbsoluteDeviation	Average deviation of the mean gray level
original_1storder_Median	Median of the gray level values in the image
Gray-Level Co-occurrence Matrix (GLCM)	
original_glcm_DifferenceAverage	Mean gray level difference of pixel pairs
original_glcm_DifferenceEntropy	Entropy of gray level differences between pixel pairs
original_glcm_DifferenceVariance	Variability of gray level differences between pixel pairs
original_glcm_Imc1	Informatics of correlation 1 for texture irregularity
original_glcm_Imc2	Informatics of correlation 2 for texture irregularity
original_glcm_InverseVariance	Uniformity of the image texture
original_glcm_JointEnergy	Uniformity and repetitiveness of the texture
original_glcm_JointEntropy	Irregularity and complexity of the texture
original_glcm_SumEntropy	Total entropy of all elements in the joint matrix
Gray-Level Run-Length Matrix (GLRLM)	
original_glrlm_LongRunEmphasis	Areas with long continuous gray levels in the image
original_glrlm_RunEntropy	Complexity of length and gray level variations
original_glrlm_RunVariance	Consistency of length variations
Gray-Level Size Zone Matrix (GLSZM)	
original_glszm_NonUniformityNormalized	Normalized non-uniformity of size zone distribution
Neighborhood Gray-Tone Difference Matrix (NGTDM) Features	
original_ngtdm_Contrast	Magnitude of local gray level differences
original_ngtdm_Strength	Strength of the image texture
original_glszm_SmallAreaHighGrayLevelEmphasis	Distribution of high gray values in small areas

For the feature selection process, as depicted in Fig. 6d, we employed a comprehensive approach to retain the most relevant and non-redundant features. Initially, features were filtered based on the Pearson correlation coefficient to identify and remove highly correlated features, reducing redundancy. Features with a high Pearson correlation coefficient (|r| > 0.9) were considered redundant, and one of each pair was removed. Following this, the mRMR (Minimum Redundancy Maximum Relevance) algorithm^52^ was applied to the remaining features, selecting features that were mutually dissimilar (minimum redundancy) but highly relevant to the target variable (maximum relevance). Finally, LASSO regression^53^ was used to further refine the feature set. LASSO applies a penalty to the absolute size of the regression coefficients, effectively shrinking some coefficients to zero. Only features with a coefficient greater than 0.0004 were retained, ensuring that the most significant features were selected for the final model. This multi-step feature selection process ensured that the final set of features was both highly relevant to the outcome of interest and minimally redundant^52–54^. The same method was applied to features extracted for different model configurations with varying habitat clusters (k = 1–10). The features retained from this selection process were then used to build SVM models for predicting PD.

### Model and statistical analysis

For model training, we utilized data from the Li-Cheng center as the training cohort. The data from the Dong-Hai center served as the external validation cohort to assess model performance. We employed a ten-fold cross-validation technique during training. To optimize the performance of the support vector machine (SVM) models, we conducted hyperparameter tuning using a grid search approach within the ten-fold cross-validation framework. The hyperparameters tuned included the kernel type (linear and radial basis function [RBF]), the regularization parameter (C), and the kernel coefficient (gamma, for the RBF kernel). Specifically, we tested linear and RBF kernels, with C values ranging from 0.1 to 100 (in logarithmic steps: 0.1, 1, 10, 100) to control the trade-off between achieving a low training error and minimizing model complexity, and gamma values ranging from 0.001 to 1 (in logarithmic steps: 0.001, 0.01, 0.1, 1) to adjust the influence of individual training samples in the RBF kernel. The optimal configuration was selected based on the highest area under the receiver operating characteristic curve (AUC) in the cross-validation folds. For the model with 5 habitat clusters, the best performance was achieved with an RBF kernel, C = 10, and gamma = 0.01, which balanced model complexity and generalization to unseen data. As depicted in Fig. 6e, model performance was evaluated using ROC, AUC, confusion matrix, and DCA curves. These metrics provided a comprehensive assessment of the model’s predictive accuracy, sensitivity, specificity, and clinical utility for PD prediction.

To validate the clinical baseline information, we performed chi-square tests for categorical variables and independent t-tests (for normally distributed data) or Mann-Whitney U tests (for non-normally distributed data) for continuous variables. These tests ensured no significant differences existed between the training and validation cohorts, upholding the dataset’s scientific rigor.

Given ten modeling experiments for habitat area generation across Clusters 1–10, we used the DeLong test to compare the models’ ROC curves. This non-parametric test, suitable for correlated ROC curves, allowed us to identify the optimal cluster number for the best model performance. By statistically analyzing ROC and AUC values from each cluster configuration, we determined the cluster setup yielding the most accurate and reliable PD predictions. This rigorous statistical analysis ensured the robustness and reproducibility of our findings.

To assess the clinical value of our SVM model for PD management, we employed DCA, which evaluates the added benefit of using the model’s predicted probabilities compared to traditional approaches^55,56^. The trained model predicts PD probabilities for patients in the validation set. We then define various diagnostic thresholds (probability cutoffs) and classify patients based on these thresholds. For each threshold, relevant metrics like true positives (correctly classified PD cases), false positives (healthy classified as PD), true negatives (correctly classified healthy), and false negatives (missed PD cases) are calculated. These metrics are used to compute sensitivity (ability to detect true PD) and specificity (ability to identify healthy cases). Finally, plotting sensitivity against false negatives (or 1-specificity) for each threshold generates a DCA curve. This curve visually depicts the trade-off between benefits (true positives) and potential harms (false positives) associated with using the model at different decision points. By analyzing the DCA curve, we can identify the optimal threshold that offers the best balance between benefits and harms for PD management in a specific clinical setting.

We further employed SHAP (SHapley Additive exPlanations) analysis to enhance model interpretability. SHAP values provide a unified measure of feature importance, enabling us to understand the contribution of each radiomic feature to the model’s predictions. This analysis is crucial for interpreting the model’s decision-making process and validating the relevance of selected features in predicting PD^57–61^.

## Data Availability

The MRI datasets analysed during the current study contain sensitive personal health information of the participants and were collected under ethical approval that explicitly requires case-by-case research agreements, individual data use applications, and institutional review board (IRB) confirmation for each new research project involving these data. This is a standard requirement of the local ethics committee (IRB protocol number: 2024:787) to ensure protection of participant privacy and compliance with national regulations on medical data handling in China. Therefore, the raw imaging data and associated clinical information cannot be made publicly available in a repository. However, the de-identified datasets (or relevant subsets sufficient to reproduce the key analyses) will be made available to qualified researchers upon reasonable individual request, subject to approval of a formal data sharing agreement and confirmation by our institutional review board. Requests should be directed to the corresponding author ( [tieqiang.li@regionstockholm.se](mailto: tieqiang.li@regionstockholm.se) ).

## Code availability

The custom code used for habitat segmentation, feature extraction, feature selection, and SVM model training/evaluation is publicly available in the GitHub repository at https://github.com/Liyuanzhe1993/Habitat-based-MRI-Radiomics-for-Enhanced-Parkinson-s-Diagnosis. The exact version of the code corresponding to the analyses presented in this manuscript has been permanently archived on Zenodo and assigned the following DOI: 10.5281/zenodo.18206677. There are no restrictions on access to the code.
